# Value of Fractional Exhaled Nitric Oxide in Diagnosing Mild Asthma Responsive to Inhaled Corticosteroids

**DOI:** 10.3390/jcm12093330

**Published:** 2023-05-07

**Authors:** Natasa Karamarkovic Lazarusic, Eugenija Kasap Basioli, Ena Tolic, Martina Dokoza, Gordana Pavlisa

**Affiliations:** 1 Outpatient Centre for Respiratory Diseases, 10000 Zagreb, Croatia; natasa.karamarkovic@yahoo.com; 2 Department of Pulmonology, General Hospital, 23000 Zadar, Croatia; eugenija.kasap@gmail.com (E.B.K.); martina.dokoza.md@gmail.com (M.D.); 3 Clinic for Respiratory Diseases Jordanovac, University Hospital Centre Zagreb, 10000 Zagreb, Croatia; ena.tolic1@gmail.com; 4 School of Medicine, University of Zagreb, 10000 Zagreb, Croatia

**Keywords:** mild asthma, fractional exhaled nitric oxide, methacholine provocation test, diagnostic accuracy, sensitivity, specificity

## Abstract

Background: Mild asthma is often characterized by normal spirometric values and a negative bronchodilation test (BDT), which makes accurate diagnosis challenging. The aim of our study was to evaluate the diagnostic accuracy of fractional exhaled nitric oxide (FeNO) in mild asthma. Methods: In adults with symptoms suggestive of asthma and normal spirometry values, BDT, FeNO, BPT and skin prick testing were performed. Patients with positive BPT started inhaled corticosteroid (ICS) therapy. Those with positive response to ICS were considered asthmatics. Results: There were 142 asthmatics and 140 non-asthmatics. No significant difference was found in BDT between the groups, *p* = 0.233. Median FeNO levels were significantly higher in the asthma group (49.5 ppb) than in the non-asthma group (23 ppb), *p* < 0.001. BPT was positive in 145 (51.42%) and negative in 137 (48.58%) patients. Positive response to ICS treatment was recorded in 142/145 (97.9%) patients. In diagnosing asthma, FeNO ≥ 25 ppb had a sensitivity of 75.4% and specificity of 47.9%. Conclusions: FeNO has insufficient sensitivity and specificity in mild asthma and the application of BPT is often necessary to establish an accurate diagnosis.

## 1. Introduction

Asthma is a common respiratory disorder which affects more than 350 million people worldwide [1]. It is a chronic disease characterized by airway inflammation, hyperresponsiveness, and remodeling [2]. The confirmation of the diagnosis is essential in order to avoid unnecessary treatment, overtreatment and/or missing other important diagnoses [3]. Mild asthma is the most common type of asthma, representing 50% to 75% of all asthma patients [4]. Although it is characterized by mild symptoms, like other types of asthma, it entails the risk of developing exacerbations that can even end lethally [5,6]. However, the diagnosis in patients with mild asthma is especially challenging. According to the Global Initiative for Asthma (GINA), in adults with respiratory symptoms typical of asthma, an increase or decrease in FEV1 of 12% and >200 mL from baseline, or a change in PEF of at least 20%, is accepted as being consistent with asthma [7]. Common asthma symptoms can also be found in other diseases that are frequent in the general population, such as allergic rhinitis, chronic obstructive pulmonary disease, and chronic cardiac failure. Besides, in mild asthma, bronchial obstruction is often not present, so a positive BDT is to be expected in a minority of patients [8]. Other options for documenting airflow limitation variability may be serial peak- flow measurement, or a significant increase in lung function after the introduction of anti-inflammatory drugs [7]. These approaches also have limitations. Diagnostic accuracy of peak-flow variability is low and requires a follow-up time of at least two weeks [9]. Monitoring of treatment response also delays diagnosis. Besides, the preferred approach to management of any disease is establishing a diagnosis before the introduction of medication, in order to avoid unnecessary treatment. An additional feature of asthma is bronchial hyperreactivity (BHR), which reflects airway inflammation and is related to the severity of the disease [10]. BHR is usually detected by a positive bronchial provocation test (BPT) [11,12]. In clinical practice, the most often-used test is the non-specific bronchoprovocation test with methacholine (BPT). BPT is of great value in the confirmation of BHR, but it is time consuming, costly, sometimes unavailable, and carries a risk of inducing severe bronchospasm [13]. Fractional exhaled nitric oxide (FeNO) is considered an indirect marker of airway inflammation. The concentration of FeNO is increased in asthmatic patients, including those with mild disease [14,15]. FeNO measurement is widely available, safe, non-invasive, and easy to perform. Many clinicians consider FeNO to be a valuable diagnostic tool for asthma.

The aim of our study was to evaluate the diagnostic accuracy of FeNO in the diagnosis of mild asthma.

## 2. Materials and Methods

### 2.1. Patients

This is a retrospective study that included 282 patients referred by family doctors for evaluation to the Outpatient Centre for Respiratory Diseases, Zagreb, Croatia, in the period between 2012 and 2015. After the initial assessment by a pulmonologist, patients presenting with clinical signs and symptoms suggestive of asthma were further evaluated. All of the included patients complained about symptoms such as dyspnoea, chest tightness, or cough and/or expectoration lasting for more than 3 weeks. The diagnostic procedure included FeNO measurement, spirometry with bronchodilator reversibility testing (BDT), BPT, and skin prick test. For all patients, chest X-rays were performed to exclude other diseases which could cause similar symptoms. Patients with well-known contraindications for BDT and BPT, including unstable coronary artery disease, cardiac arrhythmia, uncontrolled arterial hypertension, untreated hypothyroidism, or pregnancy were excluded. For patients with respiratory tract infections within 6 weeks before testing, the diagnostic procedure was postponed. Smokers were asked to refrain from smoking for 24 h before testing. No patient had previously diagnosed obstructive airway disease, nor had any previously used anti-asthma medication. Only patients whose baseline spirometric values were within normal limits were included in the study. In patients with positive BPT, anti-asthma treatment was started. They were followed-up for one year. Those in whom treatment resulted in improvement were considered to have asthma. Improvement was assessed based on the patient’s statement that their symptoms had decreased or disappeared, and that the asthma medication was helping them. The study was approved by the Ethics Committee of the University Hospital Centre Zagreb, No: 02/013 AG.

### 2.2. Pulmonary Function Tests

Pulmonary function tests were performed by experienced respiratory technicians. Spirometry was conducted using the Jaeger MasterScreen device (Jaeger GmbH, Wurzburg, Germany) in line with the recommendations of the American Thoracic Society (ATS)/European Respiratory Society (ERS) [16]. The forced vital capacity (FVC) and forced expiratory volume in 1 s (FEV1) were expressed in liters (L) and as percentage of predicted values (%). Airway obstruction was diagnosed when FEV1/FVC < 0.70 and/or FEV1 < 80% of predictive value [17]. Spirometry was performed before and 20 min after inhaled bronchodilator (salbutamol 400 µg from a pressurized inhaler). An increase of 12% and 200 mL in either FEV1 or FVC provided evidence of positive bronchodilator test (reversibility) [18].

BPT tests were performed in accordance with ATS standards [13]. Subjects inhaled aerosolized normal saline, followed by aerosolized saline containing methacholine, in doubling doses from 0.03 to 16.0 mg/mL. At each interval, subjects took five inhalations from functional residual capacity to total lung capacity, while wearing a nose clip. Three FEV1 maneuvers were measured 2.5 min after each dose interval; doubling doses were administered in this manner until either FEV1 fell by 20% of baseline or until the final dose was administered. The test was considered positive if there was a 20% fall in FEV1 from the baseline value (PC20) after inhaling methacholine stepwise to the maximum concentration (16 mg/mL). The results were characterized as: borderline BHR (methacholine concentration 4–16 mg/mL), mild BHR (methacholine concentration 1–4 mg/mL), or moderate-to-severe BHR (methacholine concentration < 1 mg/mL) [13].

### 2.3. FeNO Measurement

FeNO level was measured by a Niox Mino device (Aerocrine AB, Solna, Sweden) at a constant flow rate of 50 mL/s for 10 s in accordance with ATS/ERS recommendations [19]. FeNO was measured three times, with differences in measured values within ≤10%. The mean value of the three measurements was used as data for statistical analysis. FeNO was measured before pulmonary function and bronchoprovocative challenge testing. FeNO values were graded into the following 3 levels: low (<25 ppb), intermediate (25–50 ppb), and high (>50 ppb), following the ATS categorization [20].

### 2.4. Skin Prick Test

Skin prick testing was done per standard clinic protocol. All patients were tested with a screening panel of aeroallergens (Diater Laboratorios, Barcelona, Spain) using the following allergen extracts: Dermatophagoides pteronyssinus, Dermatophagoides farina, birch, hazel, timothy grass, rageed, mugwort, and cat and dog dander. Histamine hydrochloride (1 mg/mL) and 50% glycerol-saline were used as positive and negative controls. All tests were performed on the patients’ forearm by well-trained nurses. The results were recorded 20 min later by an experienced investigator. The skin prick test was considered positive if the test was positive for at least one tested allergen (indurate with a diameter ≥3 mm).

### 2.5. Statistical Analysis

Statistical analysis was performed with Statistica software, version 12 (Dell Inc. Inc., Tulsa, OK, USA).

The normality of distribution was assessed with the Kolmogorov–Smirnov test. Categorical data are presented as frequencies and percentages, while continuous data are presented as means and standard deviations.

Differences in numerical variables between the groups were assessed by using the t-test for independent samples and the χ^2^ test was used for assessment for differences in categorical variables. *p* values < 0.05 were considered significant. The diagnostic validity of the test was assessed by positive and negative predictive values. A receiver operating characteristics (ROC) curve was constructed to evaluate the specificity and sensitivity of individual cut-off levels of FeNO for differentiating asthmatics from non-asthmatics.

## 3. Results

### 3.1. Study Population

A total of 282 patients (mean age 35.6 ± 13.19 years) with clinical signs and symptoms suggestive of asthma participated in the study (182 (64.53%) female). All patients included in the study had normal basal spirometry values. BDT was positive in 50 patients (17.73%). Out of 282 patients, 137 (48.58%) had a negative BPT and 145 (51.42%) had a positive one. Patients with positive BPT were followed-up for one year. The patients’ responses to anti-asthma treatment were monitored. A positive response to treatment was recorded in 142 patients and they were considered asthmatics. The mean (±SD) concentration of methacholine required to cause bronchoprovocation among asthmatics was 1.53 ± 1.27 mg/mL. According to the ATS guideline categories [13], among subjects with asthma, 92 (64.79%) patients had mild BHR and 50 (35.21%) had moderate-to-severe BHR. There was not a significant difference in mean age, body mass index, gender and smoking status between asthma and non-asthma groups. Characteristics of studied patients are presented in Table 1.

The basal mean FEV1 expressed in L, and as a percentage of predicted value (%), were significantly lower (3.36 ± 0.78 L, 97% [92–107%]) in the asthma group than in the non-asthma group (3.68 ± 0.88 L, 102% [94–110%]), *p* = 0.001 and 0.015, respectively. There was no significant difference in the percentage of positive and negative BDT between the asthma group and non-asthma group. In the asthma group, 29 patients (20.42%) had a positive BDT, and in the non-asthma group, 21 patients (15%) had a positive BDT, *p* = 0.233. This gives the BDT test a sensitivity of 20.4% and a specificity of 15%.

The mean values of postbronchodilator FEV1 alone (in liters) were higher in the non-asthma group (3.88 L ± 0.92 L) than in the asthma group (3.59 L ± 0.83 L), *p* = 0.005. However, when expressed as ratios of achieved/predicted valuesi (%)were higher in the asthma group (107.3 ± 5.8) than in the non-asthma group (105.84 ± 5.03), *p* = 0.025.

At least one positive skin prick test result with tested aeroallergens was noted in 175 (62.1%) of patients. No significant difference was found in the incidence of a positive skin prick test between the asthma and the non-asthma group, *p* = 0.407. Out of 282 participants, FeNO values were low (<25 ppb) in 108 (38.3%) and increased in 174 (61.7%) of patients. Increased values were intermediate in 74 (26.2%), and high in 100 (35.46%) patients. The median FeNO value was 23 ppb in the non-asthma group and 49.5 ppb in the asthma group, with a *p* value of <0.001. Increased FeNO values were more common in the asthma group. In the asthma group, there were 107 (75.35%) patients with an increased FeNO values while in the non-asthma group, 67 patients (47.86%) had increased FeNO values, at *p* < 0.001. Values for lung function, BDT, FeNO, and skin prick testing in the asthma and non-asthma groups are shown in Table 2.

### 3.2. Sensitivity and Specificity of FeNO in Diagnosis of Mild Asthma

**Figure 1:**
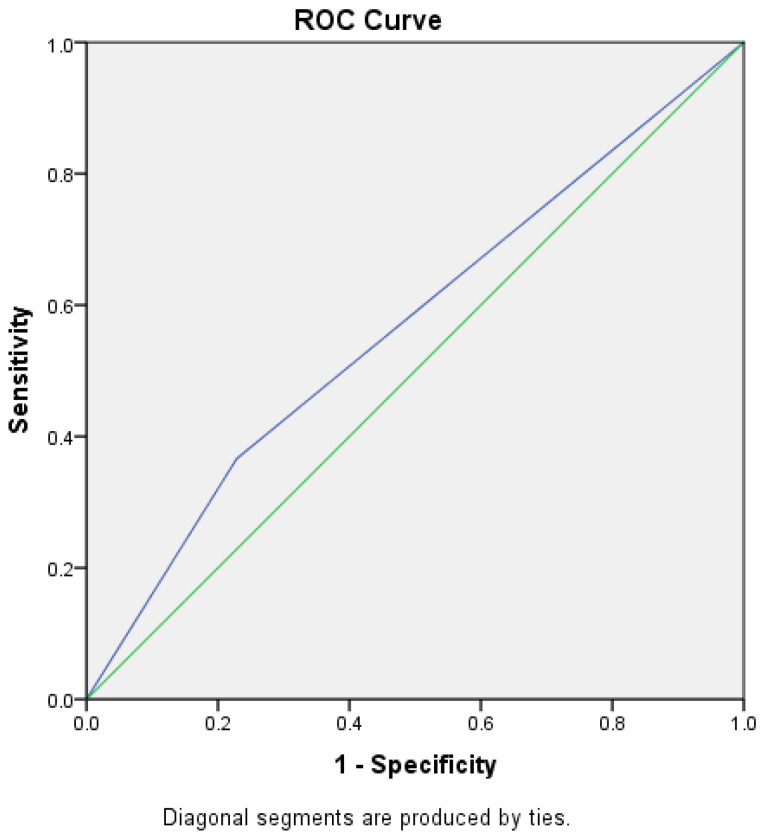
ROC curve for an FeNO cut-off value of 41 ppb. Sensitivity was 36.6%, and specificity was 22.9%.

**Figure 2:**
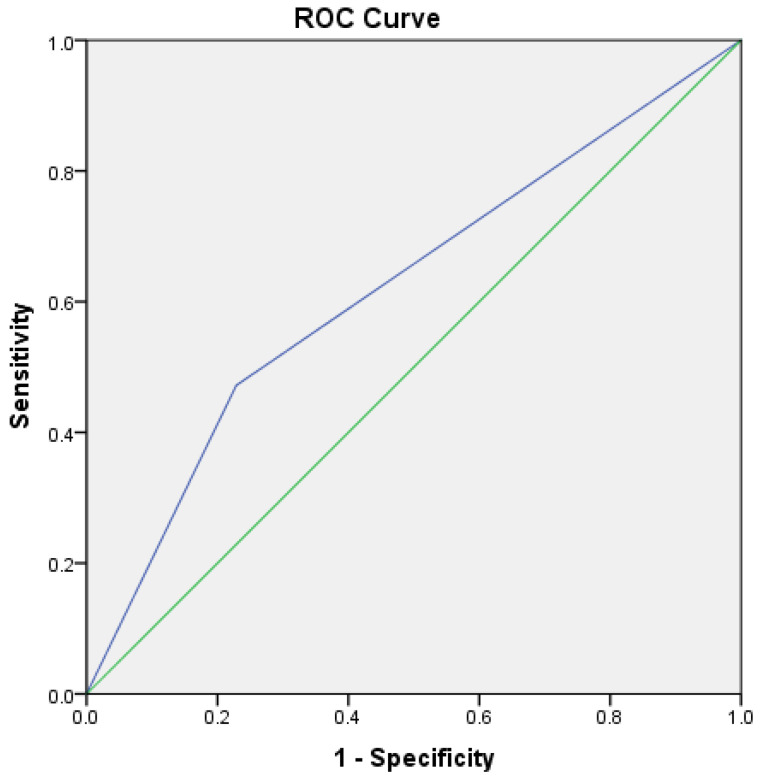
ROC curve for an FeNO cut-off value of 51 ppb. Sensitivity was 47.2%, and specificity was 22.9%.

An FeNO cut-off value of 25 ppb has sensitivity of 75.4% and specificity of 47.9% in the diagnosis of asthma. ROC curves were constructed to assess the specificity and sensitivity of higher FeNO values. At the cut-off value of 41 ppb, the sensitivity was 36.6% and specificity was 22.9% (Figure 1). At the cut-off value of 51 ppb, the sensitivity was 47.2, and the specificity was 22.9% (Figure 2). The median concentration of FeNO was significantly greater in the asthma group (49.5 ppb [25.5–81]) than in the non-asthma group (23 ppb [15–50]), *p* < 0.001.

### 3.3. Sensitivity and Specificity of BPT in Diagnosis of Mild Asthma

Of the 145 subjects who had a positive BPT, 142 subjects had a positive response to anti-asthma therapy after one year. This gives the sensitivity of BPT a value of 100% and a specificity of 97.9% in our studied population. In three patients, asthma was ruled out during follow-up. One patient was subsequently diagnosed with gastroesophageal reflux. In two patients, there was a spontaneous recovery without taking medication.

## 4. Discussion

The aim of our work was to assess the diagnostic value of FeNO in patients who were referred for pulmonological evaluation in a specialized outpatient clinic due to symptoms suggestive of asthma, but with normal basal spirometric values. The results of our studies show that FeNO is more often elevated in patients with asthma (75.35%) than in non-asthma patients (47.86%). The median FeNO value was significantly higher in the asthma group (49.5 ppb) than in the non-asthma group (23 ppb). The sensitivity of the positive FeNO test at cut-off value of 25 ppb was 75.4%, and the specificity was 47.9%. This result suggests that although many clinicians consider FeNO a valuable tool in the evaluation of asthma patients, confirmation of bronchial hyperreactivity is still necessary in establishing an accurate diagnosis.

It is very important to establish diagnosis of asthma early and accurately in order to relieve the patient’s symptoms, to prevent exacerbations and further development of chronic inflammation and airway remodeling [21]. On the other hand, over-diagnosis should be reduced as much as possible, because it is associated with unnecessary treatment and the potential risk of side effects [22]. It has been reported that, in many patients who have been diagnosed with asthma in primary care, the diagnosis cannot be confirmed later on [7]. In the study by Joyce et al., in 75% of patients who were diagnosed by primary care physicians as having asthma, bronchial hyperreactivity was not confirmed. Sixty-two percent of those patients had been treated with one or more antiasthma medications for an average duration of approximately 2 years [22]. In our study, asthma was confirmed in only about 50% of patients who presented with symptoms suggestive of asthma. For this reason, it is important to use a diagnostic method or a combination of methods that would increase the accuracy of asthma diagnosis.

International guidelines recommend that the diagnosis of asthma be based on characteristic symptoms and evidence of variable expiratory flow limitation [7]. It has been proved that clinical signs and symptoms are not specific in the diagnosis of asthma. Dyspnoea is the symptom which is most likely to cause patients to seek medical help [23]. In an epidemiological study by Bai et al., a negative correlation was found between symptoms such as dyspnoea and cough and asthma [24]. Similarly, in the study of Schneider et al., it was found that coughing and expectoration were negatively associated with asthma [25]. This is especially true for unselected patients, as is the case in primary care medicine. Many diseases have symptoms similar to asthma. Some of them are rhinitis, gastroesophageal reflux, post-viral cough, and chronic heart failure. All of them are of frequent occurrence and represent a large percentage of patients who ask for medical help in a general medical practice.

Conventional tests to confirm bronchial flow variation are of limited value, particularly among patients who have normal baseline spirometry. The most commonly-used tests are the positive bronchodilation test and confirmation of excessive variability by serial measurement of PEF. The poor sensitivity of the BDT for the confirmation of asthma has already been highlighted. This is especially the case when diagnosing mild asthma, which is often characterized by the normal basal value of FEV_1_. Significant additional bronchodilation is not to be expected in people who already have maximal bronchodilation [8]. In the studied population, the proportion of current asthmatic subjects demonstrating significant bronchodilator response was only 11.7% using a 12% FEV1 reversibility criteria [26]. In a population of suspected asthmatic patients with normal baseline spirometry, only 5.23% had post-BD FEV1 responses ≥12%. The sensitivity of post-BD FEV1 changes was 6.12%, with a high false-negative rate of 79.31% [8]. In a study of subjects with mild asthma who had normal or near-normal spirometric values, sensitivity of the bronchodilator test was 49, and specificity 70% [27]. In our study group, 17.73% had positive BDT. There was no significant difference in the percentage of positive and negative BDT between the asthma and non-asthma groups. Our results support that BDT is of low sensitivity and specificity in patients with mild asthma who initially have normal spirometry. The sensitivity of BDT was 20.4%, and it had a specificity 15%. To increase the sensitivity of the bronchodilation test, it is recommended to repeat it [7]. This approach is time-consuming and exhausting for both patient and clinician. It delays the establishment of a diagnosis, and thus delays the initiation of treatment and relief of symptoms that can often cause significant discomfort to the patients. In addition, delaying the use of anti-inflammatory drugs increases the risk of exacerbation and deterioration of lung function [28,29]. For determining BHR, a bronchoprovocation test is still considered as a reference standard, especially in cases with inconclusive spirometric results [27]. A methacholine challenge test is a highly reliable diagnostic test for airway hyperreactivity, with positive results in nearly all individuals with current symptomatic asthma [30]. Higgins et al. showed that methacholine airway hyperresponsiveness identifies twice as many subjects with physician-diagnosed asthma than does monitoring of PEF variability [31].

Since airway responsiveness is a common characteristic of asthma and is responsible for most of the clinical features of the diseases, negative BPT can rule out asthma [7]. On the other hand, excessive airway responses to nonspecific stimuli can also be detected in other respiratory entities such as allergic rhinitis or COPD [32,33]. It could also be noticed in a significant proportion of individuals with no history of respiratory diseases [34]. In our study, a positive BPT proved to be a valuable tool for confirming the diagnosis of asthma. In only three patients, asthma was ruled out during follow-up. In two patients, the symptoms regressed spontaneously, and one was subsequently diagnosed as having gastroesophageal reflux. Given that the basal spirometry in all patients was normal, the diagnosis of COPD was ruled out at the very beginning. This means that in our studied population, sensitivity of BPT was 100% and a specificity was 97.9%. The likelihood that a patient with a positive BPT has the disease increases if lower dose of methacoline causing a fall in FEV1 of more than 20% (PD20). The positive predictive value of BPT is higher in those who are more likely to have asthma based on their symptoms [13]. Our results are consistent with this finding. High sensitivity and specificity of the BPT test in our study is a result of the study design. After evaluation by a family medicine physician, all patients were examined by a pulmonologist, and those with other causes of shortness of breath (such as anemia or cardiac failure) were excluded before BPT. The test was performed only on those patients who currently had symptoms consistent with asthma. The mean concentration of methacholine required to cause bronchoprovocation in asthmatics was 1.53 ± 1.27 mg/mL. All responders reacted to a dose of methacholine up to 4 mg/mL. A total of 35.2% reacted positively to the methacholine concentration up to 1 mg/mL, and 64.8% to the concentration of 1–4 mg/mL.

Although the BPT is valuable in confirming asthma, it has significant drawbacks. BPT is time-consuming and costly, carrying a risk of severe bronchospasm and usually available only in highly specialized outpatient clinics and tertiary institutions [13]. Due to these features, it is, in general, underutilized [35].

On the other hand, FeNO tests have the advantage of being safe, repeatable, fast, simple, and easily available, and therefore more adequate [36]. Asthma is a heterogeneous disease within which several phenotypes have been recognized [7]. From a practical and therapeutic point of view, the division into type 2 and non-type 2 asthma is important. Type 2 asthma is characterized by increased production of IL-4, IL-5, and IL-13 cytokines from T helper 2 (Th2) cells promoting eosinophil recruitment and immunoglobulin production [37]. This results in eosinophilic inflammation, goblet cell hyperplasia, airway hyperresponsiveness, and immunoglobulin E (IgE) production. The main biomarkers of type 2 asthma are FeNO, blood and sputum eosinophils, and serum IgE [37]. Sputum induction helps in identifying lower-airway eosinophilia and in characterization of the asthma phenotype, but it requires specialized laboratories, is time-consuming, and it carries a risk of bronchospasm. Nitric oxide (NO) is synthesized by a family of NO synthase (NOS) enzymes. Small amounts of NO are normally produced in the lungs by a neuronal NOS and endothelial NOS, which are constitutively expressed [38]. Inducible NOS is also constitutively expressed in the human airway epithelium, but its expression is significantly upregulated in asthmatic airways, mainly in epithelial and inflammatory cells such as eosinophils, neutrophils, and macrophages [39,40]. FeNO is considered an indirect marker of IL-13-mediated type 2 airway inflammation [38]. It is estimated that type 2 asthma is present in a large proportion of children and at least 50% of adults with asthma [41]. Considering that it responds well to corticosteroid therapy, it is assumed that the exclusion of corticosteroids would reveal type 2 inflammation in an additional percentage of asthma patients [42]. Increased FeNO concentrations have been found in asthmatic patients, even in those with a mild form of the disease [14,15]. In our study, median FeNO was significantly higher in the asthma group than in the non-asthma group. Our result is consistent with the results of other authors who also showed that the median values of FeNO are higher in asthma than in other entities that have similar symptoms. Kowal et al. showed both that assessment of FeNO concentration can be used as a screening test for asthma in young adults, and its differentiation from rhinitis. The median FeNO concentration was significantly higher in the asthma group (86 ppb) than in the rhinitis/sinusitis group (37 ppb), *p* < 0.0001. A significant correlation between FeNO and bronchial hyperreactivity to methacholine was noticed [43]. Sato et al. found similar results. In the group of patients with prolonged cough, FeNO levels were significantly higher in the asthma group (93.5 ppb) than in those without asthma (16.4 ppb), *p* < 0.001 [44]. Smith et al. also found that FeNO was significantly higher in asthmatic patients than in non-asthmatic subjects with symptoms suggestive of asthma [45]. The specificity of the FeNO test in our population was 75.4%, and it had a sensitivity of 47.9%. This means that in patients with symptoms suggestive of asthma who have elevated FeNO, we could confirm the diagnosis with 75% certainty. But we also confirmed the well-known fact that the FeNO test is not specific. FeNO can be increased in various conditions such as allergic rhinitis, atopy, eczema, and eosinophilic bronchitis. However, if those diagnoses are excluded, the value of FeNO test increases. Several author groups tried to identify the optimal cut-off value of FeNO, which would differentiate asthma from other diseases with similar symptoms. A high variability of sensitivity (29% to 79%) and specificity (55% to 95%) of FeNO was observed across the studies, reflecting differences in patient inclusion criteria, given demographics such as smoking and atopy status, or concurrent ICS treatment during assessment [46]. In the group of patients with prolonged cough, the cut-off level of FeNO of 38.8 ppb had a sensitivity of 79.2% and a specificity of 91.3% in distinguishing asthma from other diseases [44]. Bao et al. showed that FENO > 41 ppb has a sensitivity of 65.29%, and a specificity of 78.16% in predicting airway hyperresponsiveness [21]. Using 40 ppb as a cut-off value for the FeNO concentration, the study of Kowal et al. proved a specificity of 82.6% and sensitivity of 88.3% [43]. We also constructed an ROC curve to assess whether higher FeNO values were more likely to predict asthma. In our studied population, even after increasing the cut-off values, we did not obtain sufficient sensitivity and specificity.

Our study has some limitations. It is well-known that the asthma phenotype affects FeNO levels. It is significantly higher in eosinophilic than in neutrophilic inflammation [7,47]. We did not analyze sputum, and therefore did not differentiate the subjects according to the type of inflammation. The vast majority of patients with mild asthma have a dry cough, so induced sputum would be needed for sputum analysis. Secondly, we collected data from patients in different seasons, which may have affected the results. This especially applies to patients with seasonal allergies. However, it can be assumed that, since the patients had symptoms when they were included in the study, they were exposed to a provoking factor, namely, a specific allergen. Due to the small number and percentage of smokers (11.7%), we did not conduct an analysis of smokers versus non-smokers, but all patients were instructed not to smoke on the day of testing.

The strength of our study may be the fact that we only included patients suspected of having asthma who presented for the first time for a diagnostic procedure and were without any asthma therapy at the moment of the diagnostic procedure. All were evaluated by a pulmonologist, and only patients with symptoms suggestive of asthma were included. Another strength of our study is that we had the possibility to follow-up on our patients and confirm the diagnosis of asthma, not only with positive methacholine testing, but also with a good response to inhaled corticosteroid therapy.

## 5. Conclusions

Although bronchial reversibility is a main hallmark of asthma, it is difficult to demonstrate in a mild form of the disease, which is mostly characterized by normal spirometric values. This explains why the BDT test is often negative and of little diagnostic value in those patients. Our results support the findings of other colleagues that FeNO, although a valuable diagnostic tool, lacks sufficient sensitivity and specificity for diagnosing asthma. BPT has high sensitivity and specificity in asthma. Our results showed that it is often necessary to perform BPT in order to establish an accurate diagnosis. Because of this, patients with mild asthma, who are generally easy to treat, often require a diagnostic procedure in the highly specialized institutions where this test can be conducted.

## Figures and Tables

**Table 1:** Characteristics of the study participants.

	Asthma (*n* = 142)	Non-Asthma (*n* = 140)	*p*-Value
Age (mean ± SD), year	34.94 ± 13.81	36.25 ± 12.58	0.405
BMI (mean ± SD), kg/m^2^	24.5 ± 4.31	25.02 ± 4.7	0.336
Gender (male/female)	57/83	43/99	0.088
Smoker/non-smoker	16/124	17/125	1.000

BMI, body mass index.

**Table 2:** Spirometric parameters, FeNO, and skin prick testing in the asthma and non-asthma groups.

	Non-Asthma (*n* = 140)	Asthma (*n* = 142)	*p*-Value
Basal FEV_1_ L (mean ± SD)	3.68 ± 0.88	3.36 ± 0.78	0.001
Basal FEV_1_% predicted, mean (range)	102 (94–110)	97 (92-107)	0.015
Post-BDT FEV_1_ L (mean ± SD)	3.88 ± 0.92	3.59 ± 0.83	0.005
Post-BDT FEV_1_% predicted (mean ± SD)	105.84 ± 5.03	107.3 ± 5.8	0.025
FeNO ppb, median (25–75 pct)	23 (15–50)	49.5 (25.5–81)	<0.001
<25 ppb *n* (%)	73 (52.14)	35 (24.65)	
25–50 ppb *n* (%)	35 (25)	39 (27.46)	
>50 ppb *n* (%)	32 (22.86)	68 (47.89)	<0.001
Skin prick positive test *n* (%)	83 (59.3)	92 (64.79)	0.407
FeNO ppb median (25–75 pct)	23 (15-50)	49.5 (25.5–81)	<0.001
<25 ppb *n* (%)	73 (52.14)	35 (24.65)	
25–50 ppb *n* (%)	35 (25)	39 (27.46)	
>50 ppb *n* (%)	32 (22.86)	68 (47.89)	<0.001
Skin prick positive test *n* (%)	83 (59.3)	92 (64.79)	0.407
FeNO ppb median (25–75 pct)	23 (15–50)	49.5 (25.5–81)	<0.001
<25 ppb *n* (%)	73 (52.14)	35 (24.65)	
25–50 ppb *n* (%)	35 (25)	39 (27.46)	
>50 ppb *n* (%)	32 (22.86)	68 (47.89)	<0.001

## Data Availability

The data presented in this study are available on request from the corresponding author.
